# Preventing Childhood Neurodisability

**DOI:** 10.21315/mjms2024.31.2.1

**Published:** 2024-04-23

**Authors:** Hussain Imam Muhammad Ismail

**Affiliations:** RCSI UCD Malaysia Campus, Pulau Pinang, Malaysia

**Keywords:** childhood neurodisability, gestation diabetes, infant nutrition

## Abstract

Globally 8.4% of children under 5 years old have a neurodisability. The important factors contributing to this are infection and inflammation, nutrition and quality of care especially during pregnancy and in the first 2 years of life. In an attempt to reduce neurodisability arising from these factors, WHO launched the 1,000 days initiative in 2014. Recent data from the National Health and Morbidity, and Malaysian National Neonatal Registry is a cause for concern. The rate of low weight babies has shown a significant increase during this period. The percentage of pregnant mothers with diabetes has doubled over the last 6 years. In addition, 20% of children under 5 years old are stunted and 46% have anaemia. All of these impact on neurological development, potentially increasing the incidence of developmental disorders and motor deficits.

## Introduction

Globally it is estimated that 8.4% of children under 5 years old have a neurodevelopmental disability (1). These children have higher levels of physical and mental health morbidity and mortality, are less likely to complete education, and are more likely to be unemployed and be socially isolated. Hence the societal costs are high. Most neurodisability is caused by events occurring early in life, specifically during pregnancy and during the first 2 years. This is a period of active brain development which sees the brain progress from the formation of the primary brain vesicles and neural tube at 4 weeks of gestation to a mature brain with a nearly fully developed cortical architecture by the end of 2 years. Recognising the vital importance of this period of human development, the WHO launched the 1,000 days initiative in 2014. This is aimed at optimising pregnancy outcomes and early child development in the hope that this will reduce the incidence of neurodisability (2).

### Malaysia and the 1,000 Days Initiative

Malaysia has always been a leader in public health initiatives and one would assume we would do well in the first 1,000 days initiative. However recent data is not very reassuring. The Institute of Public Health conducts periodic National Health and Morbidity Surveys (NHMS). The surveys in 2016 and 2022 focused on maternal and child health (3).

**Figure 1:**
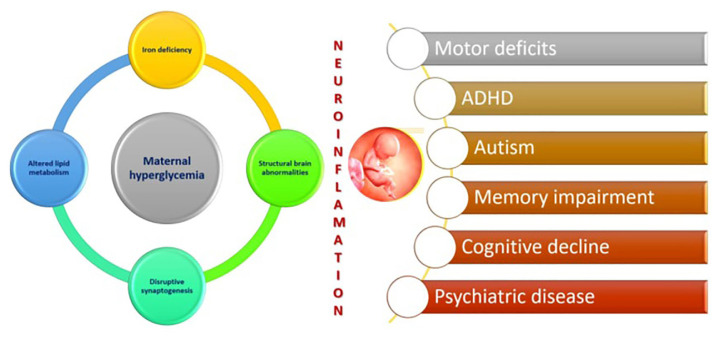
Maternal diabetes and neuroinflammation (4)

The 2022 survey revealed that the percentage of pregnant mothers with diabetes had risen to 27.1% compared to 13.5% in 2016. Maternal hyperglyceamia induces neuroinflammation that results in altered synaptogenesis. It disrupts the production of foetal monoamine neurotransmitters and brain derived neurotrophic factors causing a disruption in dendritic cell architecture. The associated abnormal lipid profile decreases the placental transfer of Omega 3 fatty acids which also impacts on monoamine neurotransmitters with consequent alterations in foetal hippocampal architecture. There is also an increase in erythropoiesis creating a relative iron deficiency in the foetus. The net result is an increase in motor impairment, cognitive disability, autism spectrum disorders (ASD) and language disability, attention deficit hyperactive disorder (ADHD) and psychiatric disorders as depicted in Figure 1 (4). This is in addition to previously well documented maldevelopment of the cardiovascular and skeletal systems as a consequence of gestational diabetes.

In brain development, neurogenesis is most prominent during early foetal development. This is followed by synaptogenesis that starts as early as 27 weeks post-conception and intensifies over the first 2 years of life following a heterochronus, cortex specific maturational pattern. This means that different parts of the cerebral cortex mature at different times between conception and 2 years of age. The hippocampus is one of the earliest parts of the brain to develop in utero, followed by the auditory cortex where maximum synaptogenesis occurs at around 3 months of age. In the prefrontal cortex it peaks at 18 months of age (5). The implication of this heterochronus pattern is that critical and sensitive periods for development, when the brain is most vulnerable to insults, are also cortex specific. This may explain why gestational diabetes particularly affects the hippocampus.

**Figure 2:**
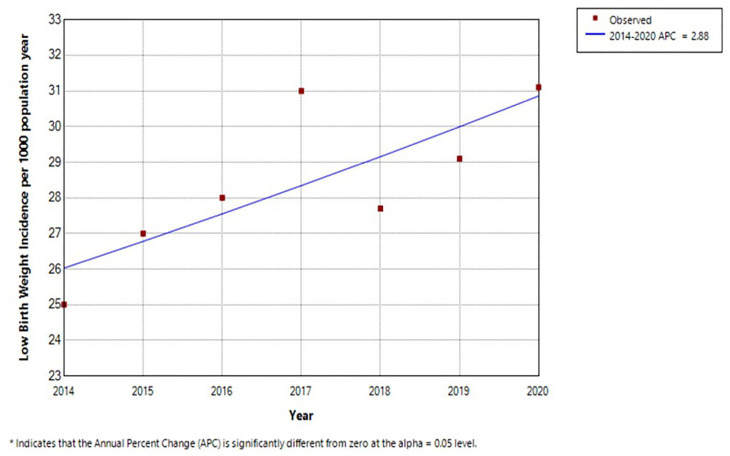
Incidence of LBW babies per 1,000 live births

**Figure 3:**
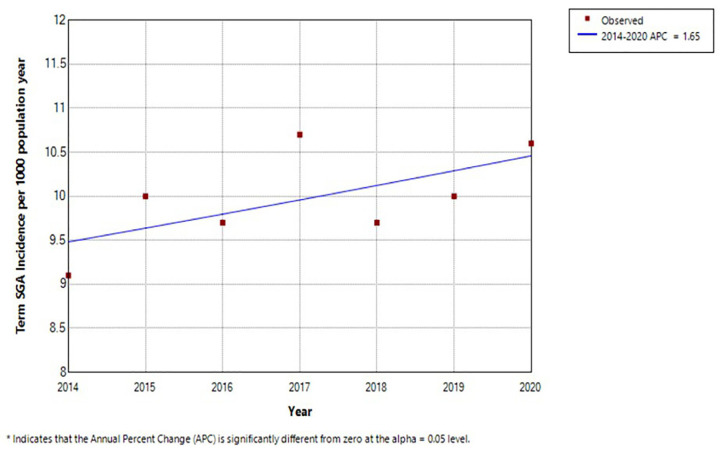
Incidence of term SGA babies per 1,000 live births

The Malaysian National Neonatal Registry has been collecting data on term and low birth weight (LBW) babies in all major hospitals since the 1990’s. Analysing data provided by the registry for the years 2014 until 2020, the incidence LBW babies shows a significant increase (*P* < 0.05) from 25.0/1,000 to 31.1/1,000 for LBW babies (Figure 2) (unpublished data). This is the opposite of the expected outcome from the first 1,000 days initiative. The largest group within this was the term 'small for gestational age' (SGA) babies. Here too was a significant increase in incidence over this period (Figure 3). Extrapolating from data collected via the MNNR to the general population, an estimated 56,423 babies born in 2020 were term SGA. A nationwide study of term SGA babies in Japan concluded that SGA in full term infants is a risk factor for developmental delay in gross motor, fine motor and behavioural domains ‘with not insignificant public health implications’ (6). This is in addition to the established poor outcome among LBW babies in general across all domains of development compared to healthy term babies.

Beyond the neonatal period, the 2022 NHMS also showed that 20% of children under 5 years old were stunted and 46% were anaemic. The most common cause of anaemia globally is iron deficiency and the brain is affected by iron deficiency before it causes anaemia (7). Iron is a key transport mediator important for the proper oxygenation and function of all tissues in the human body, including the brain. Deficiency during periods of rapid brain development are associated with dysregulation in the expression of genes critical for brain function, neurobehaviour and synaptic plasticity. Given the heterochronous nature of brain development, the impact of iron deficiency on brain development depends on whether this occurs in early or late pregnancy; in the first 6 months of life or later in infancy. This results in variable aberrations of motor, cognitive or neurophysiological development.

Recent data from the Welfare Department, Ministry of Family Affairs (Table 1) shows that of the 166,330 registered cases among children below 18 years old; 130,327 or 78% have a learning difficulty. The latter is an umbrella term that includes cognitive disability, ASD, ADHD and specific learning disorders like dyslexia. Our current understanding suggests that many of these cases could be prevented by addressing diabetes in pregnancy and nutritional deficiencies in iron and other trace elements (4, 5).

## Conclusion

In general, there are three broad factors that affect children achieving their full developmental potential: i) infection and inflammation, ii) nutrition and iii) quality of care. The first two are within the remit and responsibility of our profession. We have done well in reducing infectious causes and inflammation with one of the most comprehensive childhood immunisation programmes in the world. Now we need to address something we have taken for granted for too long, namely comprehensive antenatal care and adequate nutrition during pregnancy and the first 2 years of life.

## Figures and Tables

**Table 1:** Statistics on children with special needs registered with the Welfare Department by age and category up to 31 July 2023

No.	Age group (years old)	Visual	Hearing	Speech	Physical	Learning	Mental	Multiple	Total
1	Below 6	273	633	37	1,721	10,074	2	1,232	13,972
2	6–12	1,446	2,151	524	6,226	64,021	6	5,088	79,462
3	13–18	2,199	2,581	614	6,901	56,232	85	4,284	72,896

Source: Welfare Department, Ministry of Family Affairs (Unpublished data)
